# Finance, data and technology initiatives for noncommunicable disease control

**DOI:** 10.2471/BLT.18.220558

**Published:** 2018-12-21

**Authors:** Meri Tuulikki Koivusalo

**Affiliations:** a Faculty of Social Sciences, University of Tampere, P.O. Box 100, 33014, Tampere, Finland.

Initiatives on finance, data and technology could provide new opportunities for the prevention and control of noncommunicable diseases because they offer resources, insights from data and new monitoring means. The World Health Organization’s (WHO) independent high-level commission on noncommunicable diseases discusses the potential of these initiatives in the report *Time to deliver*, and suggests incorporating them in the commission’s future working agenda.^1^

Potential opportunities and future applications from innovative financing and public–private partnerships, data and digitalization for health have political appeal. However, the public value and relevance of these new opportunities for the prevention and control of noncommunicable diseases require further scrutiny to ensure equity in access and distribution of resources as well as long-term financial sustainability of health systems.

Here I discuss why we should also focus on analysing the risks of finance, data and technology initiatives. Such analysis requires looking into the implications of these initiatives on national policies and determining how they relate to the global norms and policy measures on control of noncommunicable diseases.

## Finance

The increase of noncommunicable diseases, especially in low- and middle-income countries, is placing a financial burden on governments and individuals. Therefore, innovative financing mechanisms for health systems are needed. Innovative financing can be sought through new taxes and levies or more private-sector-driven new financing initiatives, such as advance market commitments, bonds and public–private partnerships. However, in their report *Research and development to meet health needs in developing countries: strengthening global financing and coordination*, the WHO consultative expert working group on financing of research and development took a cautious stance to innovative financing initiatives because of the risks and costs of some of these initiatives.^2^ Reliance on private equity investors in global health and health system financing has been questioned on the grounds of similar concerns.^3^

The concerns and risks associated to these initiatives are manifold. Due to the normative role of WHO and public regulatory agencies, public–private partnerships and new models of financing can also result in conflicts of interest. While new partnerships with the private sector are often seen as vital because they could generate new resources, these partnerships are broad and can include charitable foundations, non-profit organizations or transnational industries. New financing models could have unanticipated costs, such as delays in public spending or tying public health spending to a particular treatment or policy choice. New and innovative models also tend to lack evidence on impacts; for example, evidence on social and health impact bonds is limited. 

Furthermore, public health budgets are large and may therefore prompt opportunistic business interests from corporations and investors as a new untapped resource. For example, if the World Bank or governments removed financial risk from investors, this could lead to an opportunistic engagement with the health sector, motivated by the low business risk. Another potentially problematic practice is adopting financial arrangements that allow deferred, contractual or conditional payments for health services and/or products. This could undermine legitimate and more sustainable alternative public policy measures, in particular with respect to access to costly new medicines and technologies. Policy-makers and decision-makers should thus examine innovative financing initiatives both in relation to promised health benefits and to economic risks and risk sharing, public value, accountability, costs and cost–effectiveness.

Private finance initiatives for health have led to increasing costs and concerns over the limited benefits from investment and financial market-driven approaches in public policies by nongovernmental and governmental actors. The Eurodad global report and analysis of public-private partnerships criticized the cost of such partnerships.^4^ In the United Kingdom of Great Britain and Northern Ireland, the National Auditing Office concluded that private finance procurement results in additional costs compared to publicly financed procurement, the most visible being the higher cost of finance.^5^

When governments seek to limit the costs of novel financial initiatives, contracts or partnerships with private sector and global investors, they may not realize that these initiatives might fall under bilateral trade and investment agreements. International trade and investment agreements safeguard the interests of international investors by ensuring free movement of capital. Investment agreements enable foreign investors to claim for compensation from governments through investment arbitration if governments seek to terminate or amend existing contracts in a way that breaches any of the requirements set within these agreements. These agreements can be important if governments wish to limit profits from publicly financed services. For example, a health insurance company took Slovakia to international arbitration after it sought to limit profits in publicly funded health insurance.^6^ Although not many health-related cases have been recorded yet, it is important to note that financing arrangements may be particularly conducive to such claims. According to the United Nations Conference on Trade and Development analysis, an increasing number of arbitration cases have dealt with insurances and services.^7^

Global health policy-makers and health ministries need to understand the broader context of finance and interest politics. Potential in using investment decisions more effectively for the control of noncommunicable diseases exists, as shown by experiences from environmental policies and tobacco control.^8^ However, choices should be made carefully as corporations also use corporate social responsibility programmes to access and dialogue with politicians to influence policy decisions.^9^ Therefore, governments and policy-makers must be aware of the potential conflicts of interests when partnering with the private sector and interest groups, to avoid influence from such partners on regulatory or policy decisions.

International health organizations and health ministries should consider both the potential benefits from engaging with investors and the financial market, as well as broader policy context, conflicts of interests, public value and potential costs for tax payers.

## Data and technology

The potential of data gathering, new technologies and access to a person’s health and genetic data for new treatment options is high on the political agenda. However, experiences from the commercial sector suggest that business interests are the main reasons for using data collected on consumers. The case of sale of prescription data in United States of America is one example of the predominance of commercial priorities.^10^ Increased access to more consumer and health data as such is unlikely to solve major public health problems. Analysis of large data sets will still require meaningful research questions to bring value to public health. However, access to data can play an important role in marketing, selection and targeting of consumers. This would allow businesses and insurers to select people in terms of whom to insure, employ or provide services for, and to sell data to other corporations, investors and insurers interested in this data.

New digital technologies, robotics and artificial intelligence are increasingly marketed as tools to reduce the costs of health care, yet evidence on the impacts on costs remains limited. Benefits from new technologies tend to be more limited than what is marketed, and replacing humans by artificial intelligence can also have economic, social and ethical repercussions. A crucial question is how to understand and assess the short- and long-term health impact of new technologies. Companies, clinicians and policy-makers will need a clear framework to differentiate efficacious digital products from commercial opportunism.^11^

While technological advances such as the internet and mobile phones have been crucial for communication, this progress has not necessarily led to fundamental changes in health policy. Politics of hype can distract policy-makers and the public from associated financial vulnerabilities or risks when technologies fail. Innovation is necessary, but over-emphasizing innovation as a market commodity can undermine broader research for health. High expectations in new treatments can also lead to exploiting the patients’ hopes, particularly in cases of cancer and rare diseases. New digital solutions, which have an impact on communication with patients, can improve quality of care but are less likely to provide major cost-savings for health systems. The intertwining of business interests and regulation on health is also a concern for the assessment of health technologies, which requires independence from the health-care industries.^12^

## Innovation

Access to data and knowledge can become as important for public health as access to treatment or medicines. The role of governments in regulation, financing and governing in the public interest requires innovation governance. Digitalization and use of data remain shaped by public policies, which govern innovation and how and on what basis innovations are used.^13^ If incentives for innovation result in major new monopolies or patenting in new areas, these monopolies could imply substantial costs for the health sector.

Health ministries need to understand the implications of commercial and innovation policies on the sustainability of the financing of health systems. This is particularly important for noncommunicable diseases, since in most countries, such diseases represent a high burden on health systems and health-related spending. Key issues with respect to new technologies have not changed since the 1978 Alma Ata declaration, which placed scientifically sound, socially acceptable and universally accessible methods and technology at the core of primary health care.^14^

While various interest groups tend to seek specific global financing for noncommunicable diseases, the most essential factor to control noncommunicable diseases remains that of sustainable finance and governance of health systems and public policies. Health and broader public policy priorities on how to tackle noncommunicable diseases often conflict with commercial sector or investors’ interests. The challenge is about engaging with the private sector, but also about ensuring that global action on noncommunicable diseases is driven by, and accountable to, global and national health policy priorities.
